# Trends in and relations between children’s health-related behaviors pre-, mid- and post-Covid

**DOI:** 10.1093/eurpub/ckad007

**Published:** 2023-02-01

**Authors:** Anne G M de Bruijn, Sanne Cornelia Maria te Wierike, Remo Mombarg

**Affiliations:** Faculty of Behavioural and Movement Sciences, Vrije Universiteit Amsterdam, Amsterdam, The Netherlands; School of Sport Studies, Hanze University of Applied Sciences, Groningen, The Netherlands; School of Sport Studies, Hanze University of Applied Sciences, Groningen, The Netherlands; Faculty of Behavioral and Social Sciences, University of Groningen, Groningen, The Netherlands

## Abstract

**Background:**

Covid-19 has had a major negative impact on children’s engagement in health-related behaviors. This study examines trends in children’s screen time, outdoor play and sports club membership in pre- (2016–19), mid- (2020–21) and post- (2021–22) Covid years. Also, predicting factors (gender, age and active commuting) of these health-related behaviors are examined.

**Methods:**

Data were collected via yearly self-report questionnaires among pupils in Grades 3–6 (mean age 10.14 ± 1.25 years; total *n* over the five cohorts = 6351, 50.8% girls). Multilevel path models were constructed in Mplus to examine whether children’s screen time, outdoor play and sports club membership differed between pre-, mid- and post-Covid years; and which factors predicted engagement in these health-related behaviors (using data of all cohorts).

**Results:**

During and after Covid-19, children’s screen time was higher, while they engaged less in outdoor play and were less often member of a sports club than before. Although these negative trends peaked during Covid-19, they slowly seem to be returning to pre-Covid levels in recent year. Younger children, girls and active commuters had less minutes screen time per day and played outdoors more days per week; boys and active commuters were more often sports club member.

**Conclusions:**

Although in the first year post-Covid children seem to be engaging more in health-related behaviors than during Covid-19, we still found lower levels of engagement than in the years before Covid-19, underlining the importance of early intervention to ensure an appropriate amount of engagement in health-related behaviors for all children.

## Introduction

A sufficient amount of physical activity is vital for children’s physical, socio-emotional and cognitive development.^1^ Unfortunately, Covid-19 has had a major negative impact on children’s health-related behaviors like screen time, outdoor play and sports club membership,^2–6^ on top of the already existing negative trends in these behaviors and associated physical skills.^7–10^ Although Covid-19’s impact on the world seems to be slowly decreasing, it remains unclear whether the same holds for changes in health-related behaviors associated with Covid-19. Given the detrimental impact that insufficient engagement in health-related behaviors has on children’s development,^10–16^ it is of vital importance to counteract these negative developments. For developing interventions and policy strategies, a better understanding is needed of how health-related behaviors develop over time and what their determinants are.

Main sources of physical activity for children are organized sports^12^ and outdoor play.^13^ Organized sports refers to a subset of physical activity that is led by adults, typically in clubs and organizations, constituting rules, formal practice and competition.^14^ Children participating in organized sports generally have higher levels of outdoor play^13^ and spend less time behind a screen.^15^ It is therefore worrisome that during Covid-19, children’s sport membership dropped, possibly because of fewer opportunities to actively participate at sports clubs.^3^^,^^5^ Outdoor play refers to unstructured and unsupervised play activities taking place outdoors and during leisure time. Replacing time indoors by outdoor time has been proposed as an effective strategy for increasing physical activity.^16^^,^^17^ Yet, in recent years, children are engaging less in outdoor play, amongst others because of parental concerns for safety.^18^

A key factor influencing children’s physical activity levels is the rapid rise of electronic technology.^18^ Children between 5 and 18 years spend on average 3 h and 36 min each day behind a screen (e.g. television and telephone),^19^ with an alarming increase of on average 50–60 min per day during Covid-19.^5^ These numbers are worrying, as screen time is associated with heightened risks of physical and psychological health problems.^10^

Screen time, outdoor play and sports club membership all seem to depend on child-characteristics. Studies suggest that boys have more screen time,^20^^,^^21^ are more often member of a sports club,^22^ and have more outdoor time^13^^,^^20^—although the opposite direction has also been found for screen time.^20^ In addition, children’s screen time increases,^18^ and sports club membership^22^^,^^23^ and outdoor time decrease with age.^18^ Especially when developing toward adolescence, children become less interested in outdoor play and organized sports, starting to spend more time in screen-based activities.^18^ Additionally contributing to children’s daily physical activity^24–27^ is their active commuting to school, referring to the use of active (e.g. walking and cycling) instead of passive (e.g. public transportation, car) transportation modes.^25^

This study examines trends in children’s screen time, outdoor play and sports club membership pre-, mid- and post-Covid-19; and whether these behaviors are predicted by children’s gender, age and active commuting. We expect negative trends in health-related behaviors, being most pronounced during Covid-19.^2–6^ We further hypothesize that older children have more screen time,^18^ are less often member of sports club,^22^^,^^23^ and have less outdoor play,^18^ compared to younger children, as will be the case for boys compared to girls.^13^^,^^20–23^ In addition, children who actively commute to school^24–27^ are expected to have less screen time, spend more time outside and are more often member of a sports club.

## Methods

### Design

This repeated cross-sectional study uses data of five samples (1: 2016–17; 2: 2017–18; 3: 2018–19; 4: 2020–21; and 5: 2021–22), labeled as pre-Covid (2016–19), mid-Covid (2020–21) and post-Covid (2021–22). As questionnaires were distributed yearly to all primary school pupils in Grades 3–6, samples consist of different children.

### Covid-19 in the Netherlands

Part of the data collection was conducted during the Covid-19 epidemy, during which a first lockdown was announced in the Netherlands in March 2020, ordering a nationwide closure of schools, sports clubs and public locations. From April onwards, team sports were again allowed for children. Primary schools reopened in May, although mostly allowing only half of the class present at school at the same time. Schools fully reopened in June and sports clubs in July. A second lockdown was installed in December 2020, again closing all schools and sports clubs, although children up to 17 years were allowed to play sports outsides. Between January and April 2021, a curfew was in effect. Primary schools reopened in February and sports clubs in May. In December 2021, a third lockdown closed all public locations, with schools closing 1 week earlier than the planned Christmas holidays. Children up to 12 years were allowed to play sports outside. Schools reopened after the Christmas break, and sports clubs on 15 January. Covid-19 regulations were turned in non-obligatory advices in March 2022.

### Participants

In total, 6351 children (mean age 10.14 years, SD* *=* *1.25) from 27 Dutch primary schools participated, 3224 girls (50.8%). The descriptive statistics per sample are presented in table 1. Parents provided informed consent for their child’s participation at the beginning of the school year.

**Table 1:** Descriptive statistics per sample

	Total	2016–17	2017–18	2018–19	2020–21	2021–22
*N*	6351	1821	1641	1627	426	871
Gender *n* girls (%)	3224 (50.8)	933 (51.2)	834 (50.8)	796 (48.9)	238 (55.9)	423 (50.6)
Mean age (SD)	10.14 (1.3)	10.05 (1.3)	10.15 (1.2)	10.12 (1.3)	10.13 (1.3)	10.33 (1.2)
Grade *n* pupils (%)						
Grade 3	1380 (21.6)	457 (25.1)	315 (19.2)	381 (23.4)	101 (23.7)	126 (14.5)
Grade 4	1725 (27.0)	484 (26.6)	479 (29.2)	397 (24.4)	115 (27.0)	250 (28.7)
Grade 5	1657 (25.9)	423 (23.2)	471 (28.7)	436 (26.8)	105 (24.6)	222 (25.5)
Grade 6	1624 (25.4)	457 (25.1)	376 (22.9)	413 (25.4)	105 (24.6)	273 (31.3)

### Questionnaire

For ‘screen time’, children indicated how many minutes per day they spent behind a screen, choosing from five options: <30 min, 30–60 min, 1–2 h, 2–3 h or >3 h. Previous research has shown that children’s self-reported screen time has adequate test–retest reliability (0.69–0.80^27^) and this measure of screen time has been successfully used in previous studies.^19^^,^^28^

To measure ‘outdoor play’, children indicated how many days per week they played outside. In 2016–17 and 2017–18, children were asked about the number of days per week (1–7), whereas in the last three samples, children could choose from five options (never, 1 day a week, 2–3 days a week, >3 days a week and every day). Answers of the first two datasets were recoded into the five categories of the later samples to combine data. This measure of outdoor play was adapted from Silva and Santos,^29^ who reported satisfactory test–retest reliability.

‘Sport club membership’ was measured with the question ‘Are you member of a sports club?’ which children could answer with yes or no. This measure is similar to one used and validated in a study by Moeijens et al.^30^ In this field of research, children’s sports club membership is commonly indicated by a binary categorical variable stating whether children are sport club member or not.^31^

‘Commuting to school’ was measured by asking about children’s usual mode of school travel. In the first two samples, children were asked how they traveled to school for each weekday separately. In the last three samples, children indicated how they usually traveled to school. For both questions, children had five answer options: walking, cycling, being brought (by car, on their parent’s bike), public transportation, or else—which they could elaborate upon in an open question. Answers in the first two samples were recoded into a general active or inactive transportation mode (3 days per week or more). For all samples, a variable was constructed representing active commuting (walking and cycling) vs. non-active commuting (being brought or public transportation). When assessing school commute, it is common to ask for the usual transportation mode.^27^

### Procedure

In each sample, children answered ∼50 questions about their health-related behaviors. Included topics differed per year, depending on national policies and (inter)national developments. Included questions were determined by researchers, in collaboration with teachers and the municipality. Questionnaires were sent yearly to physical education teachers at participating schools, who administered the questionnaire to all children in Grades 3–6. Children answered the questions individually on a computer in their own classroom under supervision of their teacher, taking ∼20 min.

### Analyses

Data of the different years were combined using IBM SPSS Statistics 27. Missing data analysis indicated that there were 55 cases with missing values, which were missing completely at random [Little MCAR test: *χ*^2^ (3)=7.07, *P *=* *0.07]. Missing data were handled using Full-information Maximum Likelihood in Mplus,^32^ an adequate method for handling missing data, using available data to compute a likelihood function for each participant.^33^

Following, multilevel path models were constructed in Mplus, using the Weighted Least Square Mean and Variance Adjusted estimator.^33^ School was added as clustering variable to account for the multilevel structure of the data. Model fit was evaluated using the chi square, root mean square of approximation (RMSEA), comparative fit index (CFI) and Tucker–Lewis index (TLI), with cut-offs of *P *>* *0.05, 0.08, 0.90 and 0.90 respectively.^33^

First, to analyze trends in health-related behaviors, a model was constructed using sample as a predictor of screen time, outdoor play and sports club membership, with gender and age as covariates. Next, to analyze differences in screen time, outdoor play and sports club membership in the pre-, mid- and post-Covid years, the four dummy-variables were included contrasting the post-Covid sample (2021–22) to previous samples (pre-Covid: 2016–19; mid-Covid: 2020–21). Thirdly, a model was constructed examining age, gender and active commuting as predictors of screen time, outdoor play and sport club membership. Data of all five samples were used, as results indicated that relations in the five samples were similar.

## Results

Table 2 presents the percentage of children in each category for screen time, outdoor play, sports club membership and active commuting, in total and separated by sample.

**Table 2:** Number of pupils (%) for screen time, outdoor play, sports club membership and active commuting, for the total group and separated per sample

	Total	2016–17	2017–18	2018–19	2020–21	2021–22
Screen time						
<30 min	844 (13.3)	328 (18.0)	240 (14.6)	185 (11.4)	32 (7.9)	59 (6.8)
30 min–1 h	1796 (28.2)	585 (32.1)	477 (29.1)	467 (28.7)	66 (16.3)	201 (23.1)
1–2 h	1386 (21.8)	380 (20.9)	388 (23.6)	373 (22.9)	65 (16.0)	180 (20.7)
2–3 h	1009 (15.8)	238 (13.1)	237 (14.4)	261 (16.0)	94 (23.2)	179 (20.6)
>3 h	1331 (20.9)	290 (15.9)	299 (18.2)	341 (21.0)	149 (36.7)	252 (28.9)
Outdoor play						
Never	241 (3.8)	34 (1.9)	50 (3.0)	71 (4.4)	32 (7.9)	54 (6.2)
1 day a week	437 (6.9)	88 (4.8)	82 (5.0)	158 (9.7)	41 (10.1)	68 (7.8)
2–3 days a week	1412 (22.2)	359 (19.7)	358 (21.8)	387 (23.8)	97 (23.9)	211 (24.2)
4–6 days a week	2157 (33.9)	758 (41.6)	578 (35.2)	487 (29.9)	116 (28.6)	218 (25.0)
Every day	2119 (33.3)	582 (32.0)	573 (34.9)	524 (32.2)	120 (29.6)	320 (36.7)
Sports club membership						
Yes	4584 (72.1)	1325 (72.8)	1233 (75.1)	1197 (73.6)	242 (56.8)	587 (67.4)
No	1777 (27.9)	496 (27.2)	408 (24.9)	430 (26.4)	159 (37.3)	284 (32.6)
Active commuting						
Yes	5289 (85.1)	1533 (86.8)	1361 (85.2)	1347 (84.2)	324 (78.5)	724 (86.2)
No	926 (14.9)	233 (13.2)	236 (14.8)	252 (15.8)	89 (21.5)	116 (13.8)

### Trends over time

A model in which trends in screen time, outdoor play and sports club membership were examined fitted the data well [*χ*^2^ (1)=0.019, *P *=* *0.89, RMSEA = 0.00, CFI = 1.00, TLI = 1.02]. Sample was a significant and positive predictor of screen time (*β* = 0.14, 95% CI=0.10; 0.17), and a significant negative predictor of outdoor play (*β*=−0.05, 95% CI=−0.08; 0.02) and sports club membership (*β*=−0.05, 95% CI=−0.09; −0.02). Children in more recent samples had more screen time per day, played outdoors fewer days a week and were less often member of a sports club compared to children in earlier samples, see Supplementary appendix S1.

### Health-related behaviors pre-, mid- and post-Covid-19

Secondly, we compared screen time, outdoor play and sports club membership in the samples before (2016–19) and during (2020–21) Covid-19 to the post-Covid-19 sample (2021–22). This model fitted the data well [*χ*^2^ (9)=27.53, *P *=* *0.001, RMSEA = 0.02, CFI = 0.972, TLI = 0.905].

Results indicated that children in 2016–17 (*β*=−0.32, 95% CI=−0.38; −0.26), 2017–18 (*β*=−0.20, 95% CI=−0.26; −0.15) and 2018–19 (*β*=−0.09, 95% CI=−0.16; −0.02) had less screen time than children in 2021–22, whereas children in 2020–21 (*β* = 0.44, 95% CI=0.36; 0.51) had significantly more screen time than in 2021.

In the years 2016–17 (*β* = 0.13, 95% CI=0.06; −0.20) and 2017–18 (*β* = 0.12, 95% CI=0.05; 0.18), children played outdoors more frequently than children in 2021–22, whereas in 2020–21 (*β*=−0.20, 95% CI=−0.32; −0.07) children played outdoors less frequently compared to children in 2021. There was no difference in outdoor play between children in 2018–19 and children in 2021–22 (*β*=−0.05, 95% CI=−0.07; 0.02).

In 2016–17 (*β* = 0.09, 95% CI=0.004; 0.18), 2017–18 (*β* = 0.16, 95% CI=0.08; 0.24) and 2018–19 (*β* = 0.11, 95% CI=0.05; 0.17) more children were member of a sports club than in 2021–22, whereas in 2020–21 fewer children were sports club member than in 2021–22 (*β*=−0.28, 95% CI=−0.39; −0.17).

### Predictors of screen time and outdoor play

Lastly, we examined factors predicting children’s health-related behaviors, using data of all five cohorts. A model using gender, age and active commuting as predictors of screen time, outdoor play and sports club membership fitted the data well [*χ*^2^ (1)=0.19, *P *=* *0.19, RMSEA = 0.00, CFI = 1.00, TLI = 1.02; see Supplementary appendix S2).

Gender (*β* = 0.34, 95% CI=0.26; 0.42), age (*β* = 0.20, 95% CI=0.17; 0.22) and active commuting (*β*=−0.10, 95% CI=−0.15; −0.06) were significant predictors of screen time. Boys, older children and non-active commuters had significantly more screen time.

Age (*β*=−0.05, 95% CI=−0.09; −0.02) and active commuting (*β* = 0.09, 95% CI=0.05; 0.13) were significant predictors of outdoor play. Younger children and children who had an active method for commuting also played outdoors more often. Gender was not a significant predictor of outdoor play (*β* = 0.06, 95% CI=−0.03; 0.14).

Gender (*β* = 0.24, 95% CI=0.13; 0.36) and active commuting (*β* = 0.10, 95% CI=−0.05; 0.15) were significant predictors of children’s sports club membership. Boys and active commuters were more often member of a sports club. Age was not significantly related to sports club membership (*β* = 0.01, 95% CI=−0.04; 0.04).

## Discussion

Results of our study show that in recent years, children spend less time outdoors and are less often member of a sports club compared to children in previous years, whilst having higher amounts of screen time. Covid-19 seems to have had a negative effect on children’s health-related behaviors, with a peak in screen time and dips in outdoor play and sports membership. Fortunately, these negative trends seem to be reverting in the year after Covid-19. Children seem to be playing outdoors as frequently as pre-Covid. Although screen time and sports membership have not yet returned to pre-Covid levels, numbers are substantially more positive compared to during Covid-19, with less screen time and more children being member of a sports club. Gender, age, sports club membership and active commuting were all predicting factors of children’s screen time and outdoor play. Girls, younger children, active commuters and sports club members in general had less screen time and spent more time playing outdoors.

### Trends in health-related behaviors

The negative trends in children’s health-related behaviors are in line with results of previous studies.^10^^,^^18–20^^,^^24^ Given the negative outcomes associated with high amounts of screen time,^10^ and the beneficial effects of physical activity on children’s development,^1^ these results are worrisome. Even more concerning, similar to previous results,^1–5^ children seem to have engaged less in health-related behaviors as a result of Covid-19, resulting in a peak in children’s screen time and drop in their outdoor play and sports membership during Covid-19. Fortunately, results of our study provide a more favorable picture, showing that in the first year post-Covid, engagement in health-related behaviors is more favorable than during Covid-19. Although children still have more screen time and are less often member of a sports club than before Covid-19, they generally spend less time behind a screen, have more outdoor play, and are more often member of a sports club compared to during Covid-19. Time spend playing outdoor even seems to be back to pre-Covid levels.

It should be noted that during the post-Covid year (2021–22), some Covid-measures were still in place. Although these regulations had only a minor impact on children (i.e. schools closing 1 week earlier, sports clubs closed during Christmas break), they still might have had a marginal impact on children’s daily life. It would be worthwhile to explore trends in children’s engagement in health-related behaviors now Covid-regulations are not impacting daily life anymore.

In line with previous studies,^20^ we found an inverse relation between screen time and outdoor play, indicating that excessive screen time can have a detrimental impact on children’s outdoor play. Yet, it should be noted that in our study, Covid-19 might have been a common course, as during the Covid-19 lockdowns, children followed most of their education online (thereby greatly increasing screen time), whilst simultaneously being restricted in their opportunities for outdoor play. Yet, as negative relations of screen time with other health-related behaviors (e.g. general physical activity) and overall physical health (e.g. BMI) have been found as well,^34^ targeting screen-based behaviors could be a promising road for enhancing children’s physical health and development.

### Factors related to screen time, outdoor play and sports club membership

Similar to previous studies,^18^^,^^20^ our results indicate that children spend less time outdoors and more time behind a screen as they become older, suggesting that early engagement in health-related behaviors is vital. When children grow older, they have less free time as a result of more social and academic demands, leaving little time for other leisure activities.^18^^,^^20^ Also, interest in the outdoors seems to decline as children enter adolescence, partly because of group peer pressure.^18^ Sports club membership was not related to age, contradicting findings of previous studies in which declines in sports club membership were found as children grow older. In explaining these contradicting findings, it may be that this trend becomes more pronounced as children enter adolescence and start to lose interest in and motivation for sports.^18^^,^^23^ As our sample includes few children of this age, it is likely that the decline in sports membership with age is not yet visible in our sample.

In line with previous studies,^22^ boys were found to be more often member of a sports club than girls. Whereas previous studies have been inconsistent on gender differences in children’s screen time,^18^^,^^20^^,^^21^ our results indicate that boys have higher screen time than girls. It has been suggested that gender differences are mainly driven by boys playing video games and watching/streaming videos, whereas girls are more often texting or using social media,^21^ making it of interest for future studies to also look at gender differences in type of screen time. We found no gender differences in outdoor play, contradicting previous studies reporting more outdoor play for boys.^18^^,^^20^ This might be because previous studies often measured time (in hours/minutes) outdoors, whereas we asked children to indicate how many days per week they played outside, possibly not being a sensitive enough measure to pick up differences.^35^

Following our expectations, active commuters were more engaged in health-related behaviors. These results underline the importance of encouraging active commuting methods.^27^ Although active commuting is often limited by environmental constraints,^36^ engagement in active commute may foster positive attitudes toward other health-related behaviors.^26^^,^^27^

### Strengths and limitations

Strengths of this study include the large cohort samples, measured over 5 years, and the inclusion of a range of health-related behaviors as predictor and outcome variables.

A first limitation is that we made use of cross-sectional data, meaning that we cannot derive conclusions on the causality of the relations. Bidirectional relations between health—are likely, requiring longitudinal studies to get further insight herein.

A second limitation of this study is the measure of outdoor play, asking children how many days per week they played outdoors, limiting our results for two reasons. Firstly, this rough measure of outdoor play does not provide any information on how long children played outside. Secondly, outdoor play can entail various activities, of which not all activities involve physical activity. As outdoor play is considered important for other reasons than the benefits of physical activity (e.g. exposure to nature and sunlight^11^) it is worthwhile to also consider type of outdoor activities involved. Possibly, activities do not necessarily have to promote physical activity levels in order to positively affect children’s development.^13^

Thirdly, we focused on sports club membership, not providing insight into possible differences between type of sports clubs and frequency or duration of organized sports participation. It can be expected that these numbers will have changed during and after Covid-19 as well, possibly depending on the type of sports involved (i.e. changes might be more pronounced for team sports being affected by social distancing policies compared to individual sports).

Lastly, we used self-report measures, mostly consisting of a single item. Self-reported data are heavily prone to social desirability and recall bias,^4^ and multiple-item questionnaires are seen as more reliable than single-item questionnaires.^37^ Yet, it has been argued that multiple questions with similar content can elicit socially desirable responding, resulting in systematic errors.^38^ Single-item measures are seen as a practical alternative, resulting in reliable and valid estimates of, e.g. subjective well-being, affect and exercise.^38^

### Implications

By using existing datasets of a yearly evaluation study, we were limited in the predictors that we could include in our models. Future studies could include other factors as well, particularly socioeconomic status and BMI, as children from lower socioeconomic backgrounds often have a higher amount of screen time,^39^ whilst simultaneously having fewer opportunities to engage in physical activity and sports.^40^ Also, children with higher BMIs generally have higher amounts of screen time^39^ and engage less in physical activity.^40^ In developing interventions to counteract negative trends in screen time and outdoor play, more insight into how these factors relate to health- related behaviors is of importance.

Given the negative consequences associated with excessive screen time,^10^ and the positive effects of physical activity on children’s physical, cognitive, and socio-emotional health and development,^10–16^ our results underline that making sure that all children spend time outdoors and not too much time behind a screen is of vital importance.

## Conclusion

Our study reveals negative trends in children’s health-related behaviors, with children these days spending more time on screen-based activities, spending less time playing outdoors and being less often member of a sports club. These negative trends seem to have peaked during Covid-19. Fortunately, although children still have higher screen times and are less often member of a sports club than in pre-Covid years, trends seem to be slowly reverting back to pre-Covid levels. Age, gender and active commuting were found to be important predicting factors of children’s engagement in health-related behaviors.

## Supplementary Material

ckad007_Supplementary_Data

## Data Availability

The datasets used and/or analyzed during this study are available from the corresponding author on reasonable request. Key points Over the last 5 years, children seem to be engaging less often in health-related behaviors: with children in recent years having higher screen times, spending less time outdoors and being less often a member of a sports club compared to children in earlier years. Covid-19 seems to have had a negative impact on these trends, with children having higher screen times, spending less time outdoors and being less often a member of a sports club than children in the years before Covid-19. In the first year after Covid-19, trends seem to be slowly reverting back to pre-Covid levels, with children having lower screen times, more time outdoors and higher numbers of sports membership compared to their peers during Covid-19. Gender, age and active commuting are predictors of children’s engagement in health-related behaviors. Early intervention seems vital to ensure all children are sufficiently engaged in health-related behaviors.
