# Supplementary material for: Trends in and relations between children’s health-related behaviors pre-, mid- and post-Covid
Source: Eur J Public Health. 2023 Feb 1;33(2):196–201. doi: 10.1093/eurpub/ckad007 (PMC10066467; doi:10.1093/eurpub/ckad007)

**Appendix 1. Results on predicting factors of children’s screen time and outdoor play**

**Figure 1.** *a: Screen time, and b: sports club membership, separated by gender, in percentage of children.*

**Figure 2**. *a: Screen time, b: Outdoor play, and c: sports club membership, separated by active commuting to school, in percentage of children.*


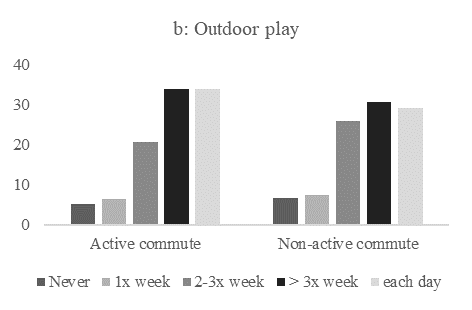

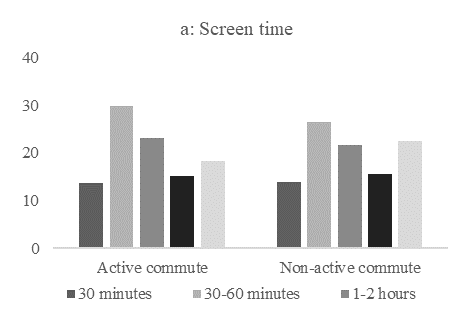

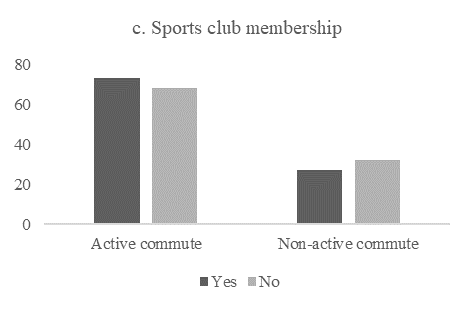


**Appendix 2.** Full model representing relations between included variables. Note: dashed lines indicate non-significant relations.


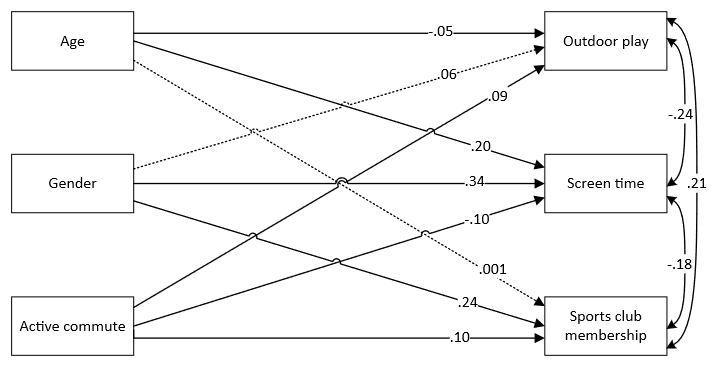

Supplement: ckad007_Supplementary_Data [file ckad007_supplementary_data.docx]
